# Acute Pancreatitis in a Young Patient With Type 2 Diabetes Mellitus Taking Sitagliptin and the Combined Oral Contraceptive Pill

**DOI:** 10.7759/cureus.105213

**Published:** 2026-03-14

**Authors:** Tharindu Ruwanpathiranage, Henry Olorunfemi, Satyanarayana V Sagi, Susan Varughese, Samson O Oyibo

**Affiliations:** 1 Acute Medicine, Peterborough City Hospital, Peterborough, GBR; 2 Internal Medicine, Peterborough City Hospital, Peterborough, GBR; 3 Diabetes and Endocrinology, Peterborough City Hospital, Peterborough, GBR; 4 Acute and Internal Medicine, Peterborough City Hospital, peterborough, GBR

**Keywords:** acute pancreatitis, combined oral contraceptive pill, dipeptidyl peptidase-4 inhibitor, drug-induced acute pancreatitis, hypertriglyceridemia, sitagliptin, type 2 diabetes mellites

## Abstract

Acute pancreatitis is a common cause of hospital admission; however, cases secondary to medication use are rare. We report a case of drug-induced mild acute pancreatitis in a 33-year-old female with type 2 diabetes mellitus. The patient presented with a one-day history of vomiting and lower chest and epigastric pain after five months of starting sitagliptin therapy. She was also taking the combined oral contraceptive pill. Clinical examination revealed epigastric tenderness and signs of dehydration. Laboratory findings showed elevated white cell count, C-reactive protein, and triglyceride levels. Although the serum amylase level was not clinically significant, a contrast-enhanced computed tomography performed 24 hours after the onset of symptoms demonstrated pancreatic inflammation and peri-pancreatic fluid consistent with acute pancreatitis. Common etiologies, including gallstones and alcohol, were excluded through imaging and history. The patient was managed conservatively with intravenous fluids for hydration and intravenous insulin for the severe hypertriglyceridemia and ketoacidosis. Sitagliptin was discontinued and replaced with gliclazide as opposed to metformin, which she was intolerant to. The combined oral contraceptive pill was continued. She demonstrated gradual improvement with normalization of inflammatory markers and triglyceride levels, achieving full recovery by day five. This case highlights the importance of considering sitagliptin and other medications, such as the combined oral contraceptive pill, as potential causes of acute pancreatitis and emphasizes the need for early recognition and prompt discontinuation of the offending agent to ensure optimal outcomes in patients.

## Introduction

Acute pancreatitis is one of the leading causes of hospital admission for gastrointestinal conditions [1]. Multiple etiological factors have been implicated in the pathogenesis of acute pancreatitis. Among the recognized causes of acute pancreatitis, gallstones account for nearly 50%, alcohol consumption accounts for 25%, and the remaining 25% are due to other causes. Drug-induced pancreatitis is relatively rare, representing approximately 0.1%-2% of reported cases [2]. Drug-induced pancreatitis is suspected when alternative etiologies have been excluded, the patient has been exposed to a medication known to be associated with pancreatitis, there is resolution on discontinuing the suspected drug, and, if possible and safe, evidence of a positive rechallenge test [2].

Sitagliptin is an oral dipeptidyl peptidase-4 (DPP-4) inhibitor commonly used to manage type 2 diabetes mellitus. Sitagliptin prolongs the duration of active incretin hormones such as glucagon-like peptide 1 (GLP-1), which is secreted by the gut, in response to food intake. GLP-1 stimulates the synthesis and release of insulin from pancreatic beta cells and reduces the release of glucagon from pancreatic alpha cells. These actions contribute to lowering the blood glucose levels [3]. DPP-4 inhibitors are generally used as second-line therapy when metformin fails to achieve diabetes control, as first-line therapy when metformin is not tolerated or is contraindicated, and in combination, dual- or triple-therapy with other oral anti-diabetic agents, including metformin.

Dipeptidyl peptidase-4 inhibitors are a recognized but uncommon cause of drug-induced acute pancreatitis. DPP-4 inhibitor-induced pancreatitis has been reported to occur at any point, ranging between three weeks and eight months following the commencement of therapy [4]. The US Food and Drug Administration (FDA) and the National Institute for Health and Care Excellence (NICE), UK, have recognized the rare association between DPP-4 inhibitors and acute pancreatitis [5]. And while evidence from clinical trials have been mixed, it is advised that this class of medications should be used with caution and discontinued if pancreatitis is suspected [6].

The combined oral contraceptive pill is used by millions of women worldwide as an effective and safe form of contraception. There are several reports in the literature implicating oral contraceptives as a rare cause of acute pancreatitis by inducing hypertriglyceridemia. Obesity, alcohol use, insulin resistance, familial hyperlipoproteinemia, and pre-existing hypertriglyceridemia are suggested risk factors for developing estrogen-induced pancreatitis [7]. There are more than 500 drugs implicated in drug-induced acute pancreatitis [2]. It is important to be aware of the various classes of drugs that can cause pancreatitis, as the diagnosis sometimes can be difficult or delayed. Here, we report the case of a 33-year-old female who developed mild acute pancreatitis five months after starting sitagliptin for the management of type 2 diabetes mellitus, while also taking the combined oral contraceptive pill. This incident was appropriately reported to the Medicines and Healthcare Products Regulatory Agency (MHRA), United Kingdom (Yellow Card report ID: GB-MHRA-MED-202511272203084970-JGRPW).

## Case presentation

Medical history and demographics

A 33-year-old woman presented with a one-day history of vomiting, followed by lower chest and epigastric pain. She had experienced approximately seven episodes of clear, non-bilious, and non-bloody vomiting. The subsequent lower chest and epigastric pain was described as sharp, central, and non-radiating. There was no back pain, no fever, or other significant symptoms of systemic infection. There was no recent travel or known exposure to infectious agents. Medical history included type 2 diabetes mellitus diagnosed one year prior, hypothyroidism, and gastroesophageal reflux disease. Surgical history included a lower-segmental cesarean section. Her regular medications included levothyroxine 75 mcg once daily, the combined oral contraceptive pill (Rigevidon), which was started two years prior, and sitagliptin 100 mg daily, which she started five months before presentation. She was intolerant of metformin due to gastrointestinal upset. There was no family history of dyslipidemia or pancreatitis. She was a non-smoker and denied alcohol consumption or the use of recreational drugs.

On admission, the patient appeared ill and clinically dehydrated. There was no icterus. She had a temperature of 37.9 degrees, a heart rate of 100 beats per minute, a respiratory rate of 18 breaths per minute, a blood pressure of 145/84 mmHg, and an oxygen saturation of 97% on room air. Cardiovascular and respiratory examinations were unremarkable. Abdominal examination revealed a soft abdomen with epigastric tenderness, and bowel sounds were present. There was no calf tenderness or peripheral edema, and a neurological examination was normal. She had a body mass index of 25.9 kg/m^2^.

Investigations

Initial laboratory investigations demonstrated an elevated white blood cell count and C-reactive protein level. Her initial serum amylase was normal (51 IU/L), but repeat testing five days later showed a mildly elevated level of 115 IU/L. Renal function, liver function, and coagulation profiles were normal. Autoimmune antibody screening for autoimmune pancreatitis, connective tissue disorders, and vasculitis was negative. (Table 1) Venous blood gas analysis revealed a pH of 7.31 and bicarbonate of 14.9 mmol/L. Serum ketone levels were elevated at 3.6 mmol/L on admission, with a blood glucose level of 11.6 mmol/L. These results were interpreted as a mixture of starvation ketosis and mild diabetic ketoacidosis. Serum cholesterol and triglyceride levels were markedly elevated when compared to levels from four months before this presentation (3.9 mmol/L and 4.6 mmol/L, respectively). This was a combined hyperlipidaemia picture. Blood cultures did not show any growth. Urine human chorionic gonadotropin testing was negative. A recent glycated hemoglobin value was 64 mmol/mol, indicating less than adequate diabetes control.

**Table 1:** Initial blood test results Results demonstrate mild metabolic acidosis with reduced bicarbonate levels, a low base excess, elevated glucose value, and elevated ketone levels, all suggesting mild diabetic ketoacidosis. There are also elevated inflammatory markers with normal liver and renal function tests, and normal serum lactate level, indicating the absence of any end-organ damage. There was also clinically significant combined hyperlipidaemia.

Blood Parameter	Result	Reference Range
Hemoglobin (g/L)	139	130–180
White cell count (10⁹/L)	16.6	4–11
Platelet count (10⁹/L)	408	150–400
Sodium (mmol/L)	127	133–146
Potassium (mmol/L)	4.2	3.5-5.3
Glucose (mmol/L)	1.6	<7
Venous pH	7.31	7.32-7.45
Venous bicarbonate (mmol/L)	14.9	23-29
Ketones (mmol/L)	3.6	<0.3
Base excess (mmol/L)	-10	-3 to +3
Troponin I (ng/L)	<5	<12
Creatinine (µmol/L)	36	59–104
C-reactive protein (mg/L)	32	<5
Amylase (5 days after presentation, IU/L)	115	<100
Lactate (mmol/L)	1.6	0.6–2.5
Total bilirubin (µmol/L)	04	<21
Alkaline phosphatase (IU/L)	114	30–130
Alanine transferase (U/L)	17	<33
Albumin (g/L)	44	35–50
Adjusted calcium (mmol/L)	2.18	2.2–2.6
Triglyceride (mmol/L)	10.3	<1.7
Cholesterol (mmol/L)	11.6	<5.0
Prothrombin time (seconds)	10	9.4–16.4
International Normalised Ratio	1.0	0.8-1.25
Activated plasma thromboplastin time (seconds)	29	24–36
Anti-nuclear antibodies	Negative	Negative
Neutrophil cytoplasmic antibodies	Negative	Negative
Immunoglobulin G4 (g/l)	0.77	0.00-1.30

**Figure 1:**
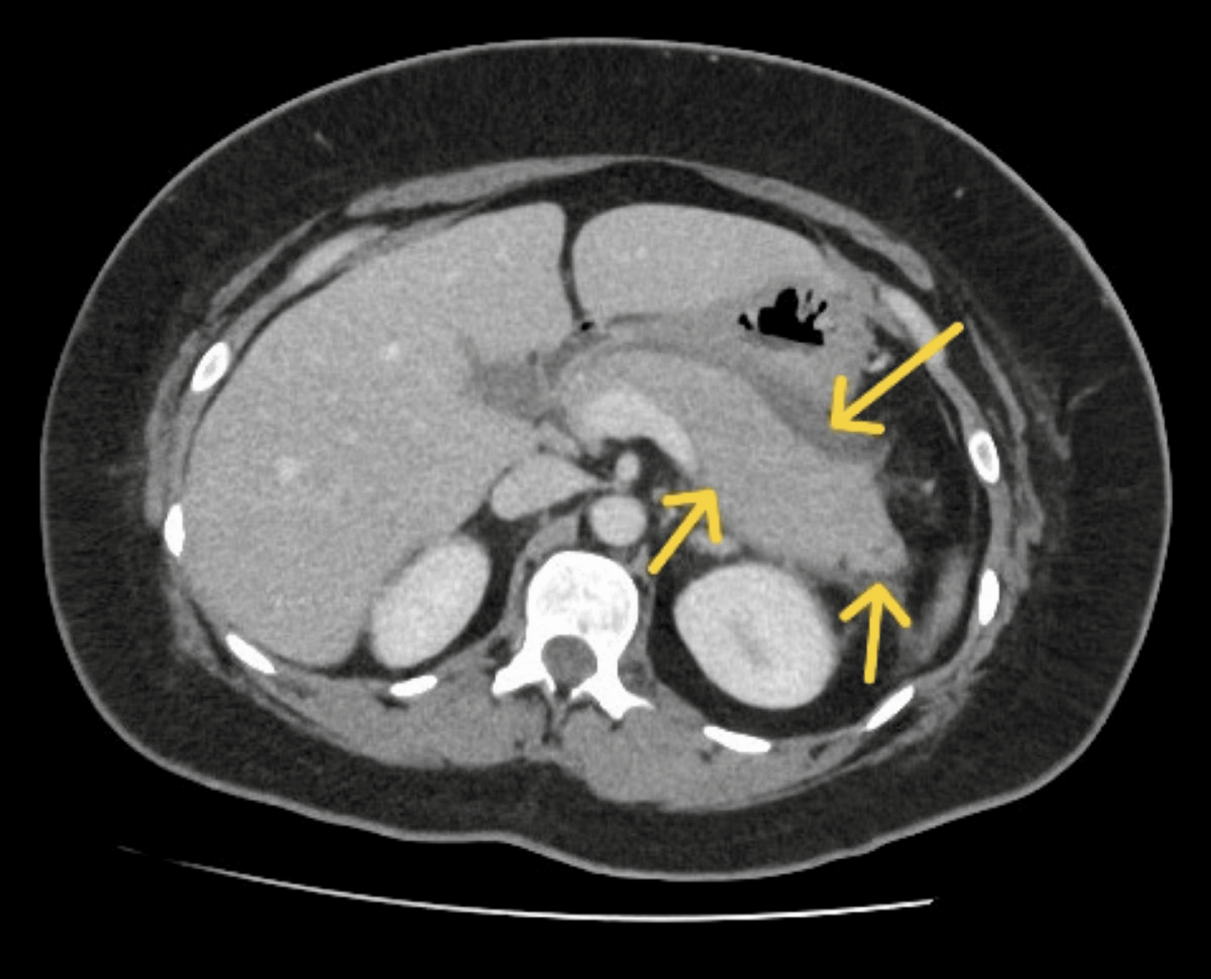
Contrast-enhanced computed tomography of the abdomen The pancreatic parenchyma is featureless and inflamed but with no necrosis. There is surrounding fluid and inflammation (yellow arrows). Features are in keeping with acute pancreatitis. No duct dilatation. No discrete collection. Normal gallbladder and biliary tree. No abnormality within the liver, pancreas, adrenals, kidneys, or bladder.

Electrocardiography demonstrated sinus tachycardia without acute ischemic or any adverse changes. Chest radiography findings were unremarkable. A contrast-enhanced computed tomography (CT) of the abdomen performed 24 hours after presentation revealed fluid and inflammation around the pancreas, consistent with acute pancreatitis (Figure 1). An abdominal ultrasound scan (USS) performed the following day revealed a thin-walled gallbladder without gallstones, and a common bile duct of normal caliber. Subsequent magnetic resonance cholangiopancreatography (MRCP) was performed to exclude congenital ductal abnormalities, and the result was normal.

Treatment

The patient was initially managed with intravenous fluids for rehydration and an insulin infusion for the hypertriglyceridemia and mild diabetic ketoacidosis, and was kept nil by mouth. Intravenous piperacillin-tazobactam was administered due to elevated inflammatory markers during the admission, along with venous thromboembolism prophylaxis. The sitagliptin was discontinued. Her blood glucose levels remained stable while on the insulin infusion. On recovery, oral feeding was cautiously reintroduced, after which insulin infusion was discontinued. Oral gliclazide 40 mg twice daily was started, as she was reluctant to use insulin due to needle phobia, and she was intolerant to metformin. Her oral contraceptive pill was continued. She also received counseling regarding dietary and lifestyle modifications to achieve long-term glycemic and metabolic control.

Outcome and follow-up

The patient demonstrated gradual clinical improvement over the following days. Serial monitoring showed significant improvement in ketone levels and inflammatory markers, with normalization of serum cholesterol and triglyceride levels (Table 2). By the sixth day of admission, the patient was clinically stable and was discharged on atorvastatin 40 mg daily as the drug of choice for combined hyperlipidaemia. Three months after discharge, her serum cholesterol and triglyceride values remained stable at 2.2 mmol/L and 2.1 mmol/L, respectively.

**Table 2:** Trend table for recovery of inflammatory markers during admission The trend data demonstrate improvement of serum cholesterol, triglyceride, and ketones by day 5 of admission. The white cell count and C-reactive protein values went up on day 3 and were back to normal by day 5 of admission. (-) test not done

Admission day	Day 2	Day 3	Day 4	Day 5	Day 6
C-reactive protein (mg/L)	-	295	311	-	76
White cell count (10⁹/L)	-	15.5	14.5	-	10.1
Cholesterol (mmol/L)	-	5.0	-	3.6	-
Triglyceride (mmol/L)	-	2.3	-	2.6	-
Ketone (mmol/L)	1.5	0.9	0.5	0.2	0.2

## Discussion

We report the case of a young female who developed mild acute pancreatitis five months after starting sitagliptin for the management of type 2 diabetes mellitus. She had also been taking the combined oral contraceptive pill for two years before presentation. Despite the supporting symptoms and imaging findings, the patient's serum amylase was not clinically significant. The patient had no other cause apart from drug-induced acute pancreatitis. The acute pancreatitis was classified as mild because there was no organ failure, no local or systemic complications, and the condition resolved within a week (Revised Atlanta Classification) [8].

According to the National Institute for Health and Care Excellence (NICE) guidelines, acute pancreatitis is diagnosed when at least two of the following criteria are met: sudden-onset abdominal pain, serum amylase or lipase levels exceeding three times the upper limit of normal, and imaging findings consistent with pancreatitis on contrast-enhanced CT [9]. In our patient, the diagnosis was based on the sudden-onset abdominal pain and imaging findings consistent with acute pancreatitis on contrast-enhanced CT. Her serum amylase level was not clinically significant. Serum amylase levels can be normal in 20-30% of individuals with acute pancreatitis, especially in those with alcohol-related pancreatitis, in the presence of hypertriglyceridemia affecting the laboratory analysis, pancreatic necrosis, and in those with delayed diagnosis. Serum lipase measurement is a more valuable test in these situations [10]. The serum lipase was not measured in this case.

Our patient started sitagliptin five months before presentation. Several case reports suggest that sitagliptin-induced pancreatitis commonly occurs within a few weeks to several months of commencing treatment but can occur at any point during treatment [4,11-14]. The mechanism for DDP-4 inhibitor-induced pancreatitis remains unclear. It has been hypothesized that the increased activity of GLP-1 results in exocrine and alpha-cell hyperplasia, increased pancreatic duct turnover, and metaplasia. This data, provided by rat studies, has strengthened the potential connection between DDP-4 inhibitor treatment and pancreatic events, such as acute or chronic pancreatitis and pancreatic cancer [15].

Our patient started the estrogen-containing combined oral contraceptive pill two years before presentation. Estrogens increase liver synthesis of triglycerides and also reduce the clearance by suppressing hepatic lipase activity. The combined oral contraceptive pill can rarely cause severe hypertriglyceridemia resulting in hypertriglyceridemic pancreatitis [7]. Of the possible risk factors (obesity, alcohol use, insulin resistance, familial hyperlipoproteinemia, and pre-existing hypertriglyceridemia), our patient did have diabetes and pre-existing mild hypertriglyceridemia. Estrogen-induced hypertriglyceridemic pancreatitis usually occurs within a few months of starting the combined oral contraceptive pill, but there are reports indicating that spontaneous severe hypertriglyceridemia can occur at any time while taking the oral contraceptive pill, followed by rapid recovery once the pill is stopped [16,17]. Serum triglyceride levels are usually above 11.3 mmol/L, and often exceed 22.6-33.9 mmol/L. Our patient’s triglyceride value did not go above this threshold, and we did not discontinue the oral contraceptive. The patient’s triglyceride levels remained controlled. Though we did not believe that the combined oral contraceptive pill was the culprit in this case, the potential for it to be a co-contributor to acute pancreatitis cannot be completely ruled out in this case.

Transient hypertriglyceridemia with mild to moderate elevations in serum triglycerides (2-10 mmol/L) is common in the initial phase of acute pancreatitis, irrespective of etiology. In one study, such elevations were observed in 47% of unselected patients presenting with acute pancreatitis. It is characterised by rapid resolution during the recovery and probably the fasting phase of acute pancreatitis. These triglyceride elevations were considered more likely to represent an epiphenomenon of the acute inflammatory process rather than a primary causal precipitant [18]. Our patient had a similar pattern of transient hypertriglyceridemia, probably secondary to sitagliptin-induced acute pancreatitis.

Our patient developed mild acute pancreatitis five months after starting sitagliptin, and two years after starting the combined oral contraceptive pill. There was no evidence of gallstones, alcohol use, or other common etiologies. She did have pre-existing mild hypertriglyceridemia, but no family history of such. The pancreatitis was accompanied by transient hypertriglyceridemia. Symptoms did not return after discontinuing sitagliptin and continuing the oral contraceptive pill. Using the World Health Organization-Uppsala Monitoring Center (WHO-UMC) system for standardized case causality assessment, the causal relationship between the administration of sitagliptin and the onset of acute pancreatitis in this case would be regarded as possible. This was the same for the oral contraceptive pill [19].

The Naranjo Adverse Drug Reaction Probability Scale is a validated 10-question questionnaire used to determine the likelihood that an adverse drug reaction is caused by a specific medication rather than other factors. It categorizes reactions as definite (≥9), probable (5-8), possible (1-4), or doubtful (≤0) [20]. Using this scale, a score of +3 was given for both sitagliptin and the oral contraceptive pill, indicating that it is possible that the acute pancreatitis was an adverse reaction to any of those medications (Table 3).

**Table 3:** The questions used in the Naranjo Adverse Drug Reaction Probability Scale The table demonstrates scores of +3, which indicate that it is possible that the acute pancreatitis occurred as an adverse reaction to either sitagliptin or the combined oral contraceptive pill. Even though sitagliptin was discontinued while the combined contraceptive pill was continued, we could not say 'yes' to question number 3 since mild pancreatitis is known to recover rapidly with conservative treatment anyway. Naranjo et al. [20].

Questions	Sitagliptin	Combined oral contraceptive pill
1. Are there previous conclusive reports on this reaction?	Yes [+1]	Yes [+1]
2. Did adverse event appear after the suspected drug was given?	Yes [+2]: started 5 months prior	Yes [+2]: has been on this for two years without any issues
3. Did the adverse reaction improve when the drug was discontinued or a specific antagonist was given?	Not known or not done, as mild pancreatitis usually recovers promptly with conservative management	Not known or not done, as mild pancreatitis usually recovers promptly with conservative management
4. Did the adverse reaction appear when the drug was readministered?	Not known or not done	Not known or not done
5. Are there alternative causes that could have caused the reaction?	Yes [-1]	Yes [-1]
6. Did the reaction reappear when a placebo was given?	Not known or not done	Not known or not done
7. Was the drug detected in any body fluid in toxic concentrations?	Not known or not done	Not known or not done
8. Was the reaction more severe when the dose was increased, or less severe when the dose was decreased?	Not known or not done	Not known or not done
9. Did the patient have a similar reaction to the same or similar drugs in any previous exposure?	Not known or not done	Not known or not done
10. Was the adverse event confirmed by any objective evidence?	Yes [+1]	Yes [+1]
Total score	+3	+3

We chose sitagliptin over the combined oral contraceptive as the primary cause because: (1) the sitagliptin was started within the prior five months while the contraceptive pill was started two years prior, (2) the transient hypertriglyceridemia was not characteristically severe as in reported cases of estrogen-induced hypertriglyceridemic pancreatitis, and (3) symptoms have not returned and serum lipids remained near-normal at 3-months follow-up after stopping the sitagliptin and continuing the oral contraceptive pill. The true causality cannot be proven without a formal drug de-challenge and rechallenge test and large population studies. 

## Conclusions

Although sitagliptin and the combined oral contraceptive pill are generally safe and effective for managing type 2 diabetes mellitus and providing effective contraception, respectively, clinicians should be aware of their rare but potentially serious association with acute pancreatitis. In patients presenting with acute pancreatitis, the medication history, including the use of sitagliptin and the combined oral contraceptive pill, should be carefully reviewed. Early recognition and prompt discontinuation of the offending agent are essential to prevent complications and ensure optimal patient outcomes.
